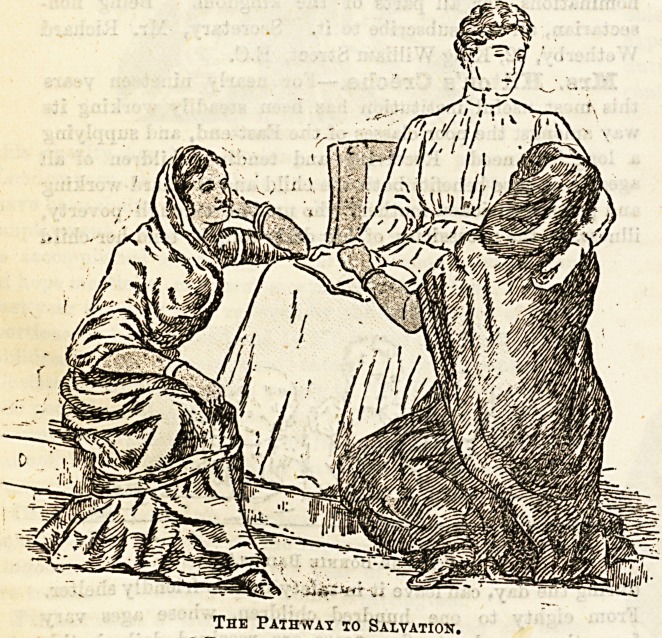# A Few Other Charities

**Published:** 1891-01-03

**Authors:** 


### A FEW OTHER CHARITIES.

**Field Lane Refuges and Ragged Schools,** Vine Street, Clerkenwell Road, E.C.—For nearly half a century

A Pathetic Group.

this charity has been working amongst the poorest of the London poor, the result being that upwards of 40,000 persons have been sheltered, and nearly 10,000 have been helped to employment. Amongst other means by which the good work is accomplished are a ragged church, mission services, band of hope meetings, mothers' meetings, and a crêche. During last year the poor were relieved by the distribution of 23,407 portions of broken food and 14,576 loaves of bread. 11,025 children's hot dinners were eaten on the premises. A Christmas dinner is given yearly to 700 of the homeless, to 500 poor deserving families, and to 700 poor children. The committee urgently appeal for funds, as these operations cannot be carried on without an increase. Secretary, Mr. Peregrine Platt.

**City of London Truss Society,** 35, Finsbury Square.—For the relief of the ruptured poor throughout the kingdom. Nearly 10,000 in the year of both sexes and ages are treated. Secretary, Mr. John Whittington.

**Fishermen's Shelter, Valencia.**—When all benevolent people are considering the claims of various great hospitals, asylums, &c., it may not be out of place to put in a plea for a community of hard-working fishers. A large fleet of fishing craft frequents the coast of Ireland during the months of March to July, the crews drawn from all parts of the United Kingdom. Their boats put into Valencia, and so 400 men are obliged by the nature of their work to be on shore many days together, but there is no place where they can shelter, except in the public-houses, on the island. It is most earnestly desired to build a suitable room and hall where they can spend those days in comfort. Only £90 is required, £210 having already been collected. Any donations will be thankfully received by the Rev. A. Delap, Incumbent of Valencia, Killenlagh, Co. Kerry.

**Home for Little Boys,** Farningham, Swanley, Kent.—An institution whose object is to receive all little lads, whether orphan or not, who, being homeless and destitute, are likely to fall into crime from lack of a friendly hand to save them. About 500 boys are provided for by this institution in their homes at Farningham and Swanley, where they are trained from an age when the mind is most impressionable to good or evil influences, till they become sufficiently expert at one of the many trades taught here to enter on life's duties fully equipped for their part in it. A greater benefit could not be conferred on any lad than to give him the training, education, and industrial instruction which changes a possible criminal into a useful and self-reliant worker. One house at Farningham is still closed for want of necessary funds, and we commend this institution to the notice of those who have little lads of their own. Secretary, Mr. B. Clarke, St. Bride Street, Ludgate Circus, E.C.

**London Orphan Asylum, Watford.** — The managers of this asylum appeal urgently for aid this year to meet their expenses, which have been unusually heavy, and have obliged them to reduce the numbers of children to be elected from forty to thirty. As this institution is dependent for nine-tenths of its income upon voluntary subscriptions, we trust that it may meet with the cordial recognition it deserves. It is specially designed for the relief and support of fatherless children of either sex, who are placed in the sad position of having once had, during the father's lifetime, a comfortable home, though now left orphans without adequate means of support. The children that seek its shelter come from all parts of the British dominions. Five hundred and sixteen are now under its parental care, and as they are drawn from all classes of society, so should all classes generously contribute in answer to their urgent appeal. Secretary, Mr. James Rogers, 21, Great St. Helen's, E.C.

HEALTH AND EXERCISE.

**National Health Society.**—The basis of the whole system of instruction which this most useful society comprises, may be said to lie in the proverb that "Prevention is

HELPFUL AND ACCOMPLISHED.

better than Cure." It goes straight to the root of the matter, as its aim is to diffuse by all possible means sanitary knowledge of every kind, comprising home nursing, rearing of infants, prevention of the spread of infectious diseases, and by no means least, important lectures on sanitary reform in food and cookery. The society gives courses of lectures on the various subjects which affect health, not only in London and its vicinity, but even so far afield as Glasgow and Aberdeen. We wish this useful society all success in its work. Secretary, Miss Lankester, 53, Berners Street, W.C.

**Metropolitan Drinking Fountain and Cattle Trough Association.**—The relief afforded to both human beings and dumb animals by this Society is incalculable, as it is the only one which provides free supplies of water for man and beasts in the streets of London. Secretary, Mr. M. W. Milton, 111, Victoria Street, Westminster, S.W.

**Religious Tract Society.** — For 88 years has this Society continued to bear a large share of the work of spreading the Gospel in all parts of the world. Funds are urgently needed, and will gladly be received by the Secretaries, Rev. L. B. White, or Rev. S. Green, 56, Paternoster Row, E.C.

**Royal Albert Orphan Asylum,** Bagshot, Surrey. — A noble work is undertaken by this charity, viz: the clothing, feeding, and education of destitute orphan children of all denominations, for all parts of the kingdom. Being non-sectarian, all can subscribe to it. Secretary, Mr. Richard Wetherby, 62, King William Street, E.C.

**Mrs. Hilton's Crêche.**—For nearly nineteen years this most useful institution has been steadily working its way amongst the poor classes of the East-end, and supplying a long-felt need. Receiving and tending children of all ages, it at once benefits both the child and the hard-working and poverty-stricken mother, who unable, through poverty, illness, or the necessities of her daily toil, to tend her child

"THREE BONNIE BAIRNS."

during the day, can leave it in safety in this friendly shelter. From eighty to one hundred children, whose ages vary from three weeks to five years, are received daily in this crêche and infirmary, where they are washed, clothed, fed, and tended for twelve hours each day. Mrs. Hilton's Crêche, whilst it relieves the parent of the burden of its care, lays the foundation of better health in the child by providing good food and careful nursing. All contributions are earnestly desired, and should be sent to Mrs. Hilton, 12, Stepney Causeway, E.

**Ragged School Union.**—Serving as it does as a central bond of union for ragged schools, missions, and kindred agencies among the very poor in London, and throughout the United Kingdom, we are, sure our readers

All Sorts and Conditions.

will be willing to assist this charity either by a donation, or subscription, or by gifts of old garments, material, books, or by making its objects more widely known. Secretary, Mr. John Kirk, Exeter Hall, W.C.

**Zenana, Bible, and Medical Mission.**—This society has a threefold object in its work, addressing itself to the spiritual, mental, and bodily evils under which the unhappy women of India have groaned for centuries. It is worthy of help from all. Especially should it appeal to women, for their sisters of India can only be reached by women; their sufferings only alleviated by women; and only women can enter their homes and personally influence them in mind and body. During the last year at the hospitals and dispensaries

The Pathway to Salvation.

at Lucknow and Benares, 18,782 attendances were registered and 5,663 patients. A hospital is urgently needed at Patna to carry on the good work, and so energetic is the society in its catholic and unsectarian labours, that we hope the generosity of English men and women will enable them to found this much-needed institution, and also to send out what is so urgently desired, six additional lady workers, to occupy centres of immense population, and carry the light of medical skill and education into the dark places of the earth.

**Royal Society for the Prevention of Cruelty to Animals.**—To all animal lovers it is unnecessary to commend this Society, its work is so well known and its benefits so obvious. Its influence extends over the entire Kingdom, and every one must heartily wish well to an institution to which the present more merciful treatment of animals is entirely due. To diminish in the slightest degree the far-reaching efforts of this Society would be a truly national loss; but we much regret to hear that unless liberal support is at once obtained this excellent work must be abridged. Secretary, Mr. John Colam, 105, Jermyn Street, S.W.

---

The following are deserving institutions, to the work of which we would also call the attention of the charitable:—

### General Hospitals.

German Hospital, Dalston, E.—Secretary, Mr. C. Feldmann; Matron, Miss Christiane Burger.

London Homœopathic Hospital, Great Ormond Street, W.C.—Secretary, Mr. G. A. Cross; Lady Superintendent, Miss Brew.

London Temperance Hospital, Hampstead Road, N.W.—Secretary, Mr. T. Mundy; Matron, Miss S. E. Orme.

Middlesex Hospital, Mortimer Street, W.C.—Secretary, Mr. F. Clare Melhado; Lady Superintendent, Miss Thorold.

North-West London Hospital, 18, Kentish Town Road.—Secretary, Mr. Alfred Craske.

St. George's Hospital, Hyde Park Corner, S.W.—Secretary, Mr. C. L. Todd; Matron, Mrs. Coster.

Seamen's Hospital (Dreadnought) Society, Greenwich.—Secretary, Mr. P. Michellis; Matron, Miss Cooke.

University College Hospital, Gower Street, W.C.—Secretary, Mr. Newton H. Nixon.

West London Hospital, Hammersmith Road, W.—Secretary, Mr. R. J. Gilbert; Lady Superintendent, Miss Hardy.

Westminster Hospital, Broad Sanctuary, S.W.—Secretary, Mr. Sidney N. Quennell; Matron, Miss Pyne.

### Hospitals for Consumption.

Royal National for Consumption, Ventnor.—Secretary, Mr. E. Morgan, 34, Craven Street, W.C.; Lady Secretary, Miss Moore.

### Hospitals for Children.

Alexandra Hospital for Children, 18, Queen Square, W.C.—Matron, Miss Moore.

Belgrave Hospital for Children, 79, Gloucester Street, Pimlico.—Hon. Secretaries, the Rev. J. Storr and Captain W. J. Stafford; Lady Superintendent, Miss Munro.

Cheyne Hospital, 46, Cheyne Walk, S.W.—Honorary Secretary, Mr. E. W. Flower: Matron, Miss Elam.

East London Hospital For Children, Shadwell, E.—Secretary, Mr. Samuel Whitford; Matron, Mrs. Fisher.

Evelina Hospital, Southwark Bridge Road, S.E.—Secretary, Mr. T. S. Chapman; Matron, Miss Alice Cross.

Home for Incurable Children, 2, Maida Vale, W.—Matron, Miss Coleman.

North-Eastern Hospital for Children, Hackney Road, E.C.—Secretary, Mr. Alfred Nixon; Matron, Miss E. W. Curno.

Paddington Green Children's Hospital, W.—Secretary, Mr. H. W. Pearce; Matron, Miss Anderson.

Victoria Hospital for Children, Queen's Road, Chelsea.—Secretary, Captain W. C. Blount; Matron, Miss Cooper.

### Hospitals for Women.

Chelsea Hospital for Women, Fulham Road.—Secretary, Mr. A. C. Davis: Matron, Miss Wade.

Grosvenor Hospital for Women and Children, Vincent Square, Westminster.—Secretary, Hon. F. C. Howard; Matron, Miss K. Hughes.

New Hospital for Women, 322, Marylebone Road, W.—Secretary, Miss C. Vincent; Matron, Miss Margaret M. Bagster.

Samaritan Free Hospital, 13, Lower Seymour Street, W.—Secretary, Mr. George Scudamore; Matrons, Miss Butler and Miss Tidy.

### Cancer.

Cancer Hospital, Brompton.—Secretary, Mr. W. H. Hughes; Matron, Miss A. Rogers.

### Hospital for Incurables.

British Home for Incurables, Clapham.—Secretary, Mr. R. G. Salmond, 73, Cheapside, E.C.

### Miscellaneous Special Hospitals.—Dental.

Dental, Leicester Square, W.C.—Secretary, Mr. J. F. Pink.

National Dental, 149, Great Portland Street.—Secretary, Mr. Arthur G. Klugh.

### Diseases of the Throat.

Hospital for Diseases of the Throat, Golden Square, W.—Secretary, Mr. W. Thornton-Sharp; Matron, Miss M. B Mackey.

### Ophthalmic.

Central London, 238, Gray's Inn Road.—Secretary, Mr. William Abrams; Matron, Miss Marian Hardwick.

Royal London, Blomfield Street, E.C.—Secretary, Mr. A. J. Newstead; Matron, Miss Nichol.

Royal South London, St. George's Circus, S.E.—Secretary, Mr. Charles Comyns.

Royal Westminster, King William Street, W.C.—Secretary, Mr. T. Beattie-Campbell.

Western, 153, Marylebone Road.—Secretary, Mr. G. T. Manton.

### Orthopædic.

City Orthopædic, 27, Hatton Garden, E.C.—Secretary, Mr. Ernest Derenth; Matron, Miss Pollard.

National Orthopædic, 284, Great Portland Street, W.—Secretary, Mr. Hole; Matron, Miss F. W. Quesidder.

Royal, 297, Oxford Street, W.—Secretary, Mr. Benjamin Maskell; Matron, Mrs. Willicombe.

## Figures and Tables

**Figure f1:**
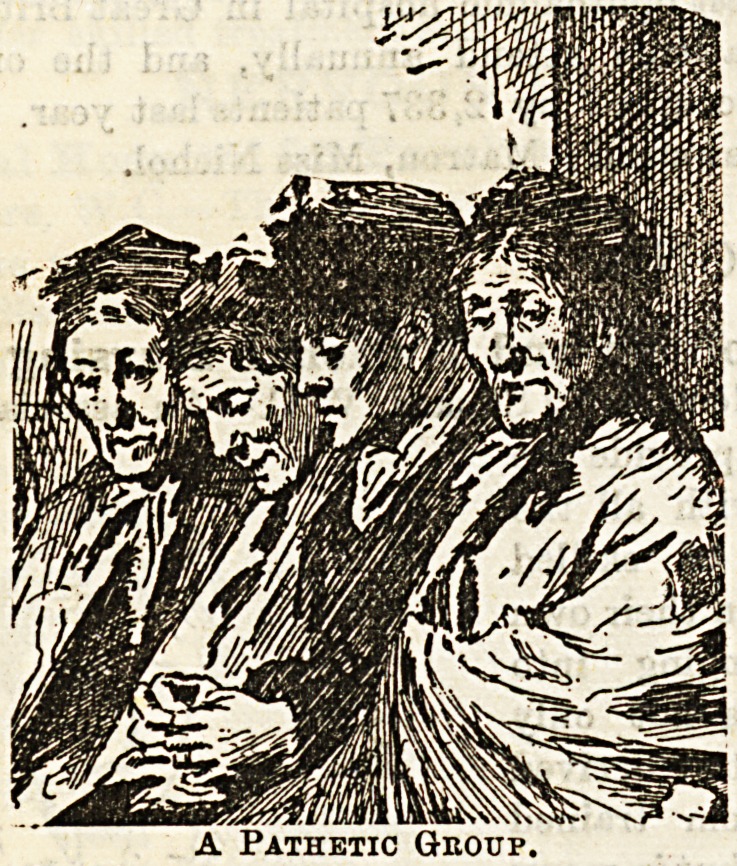


**Figure f2:**
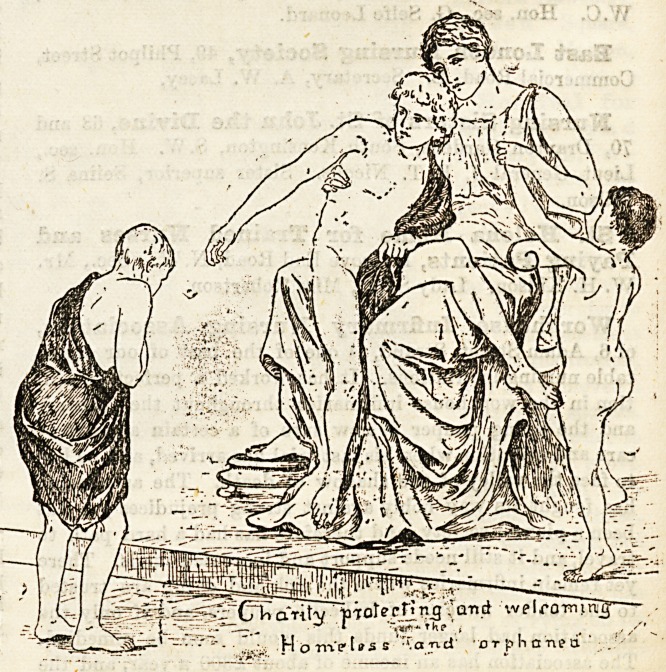


**Figure f3:**
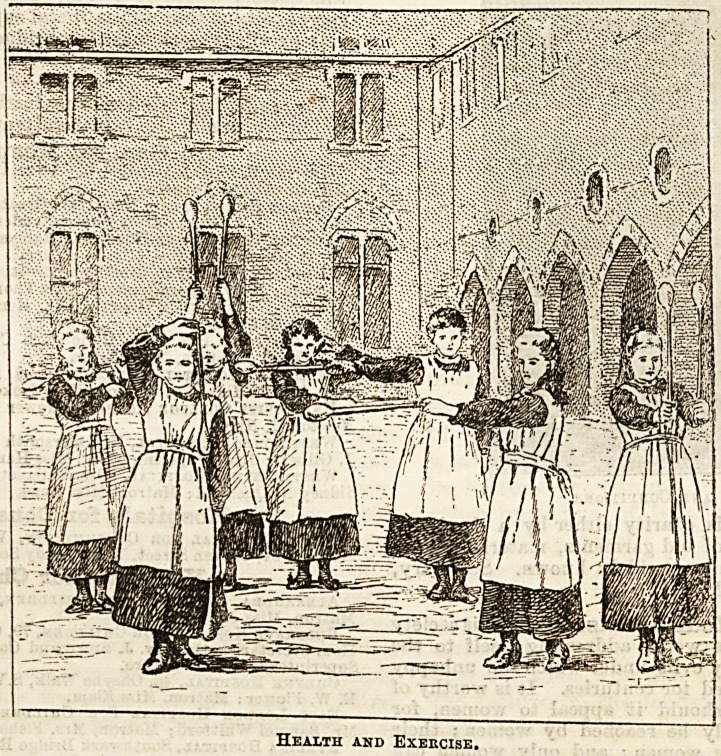


**Figure f4:**
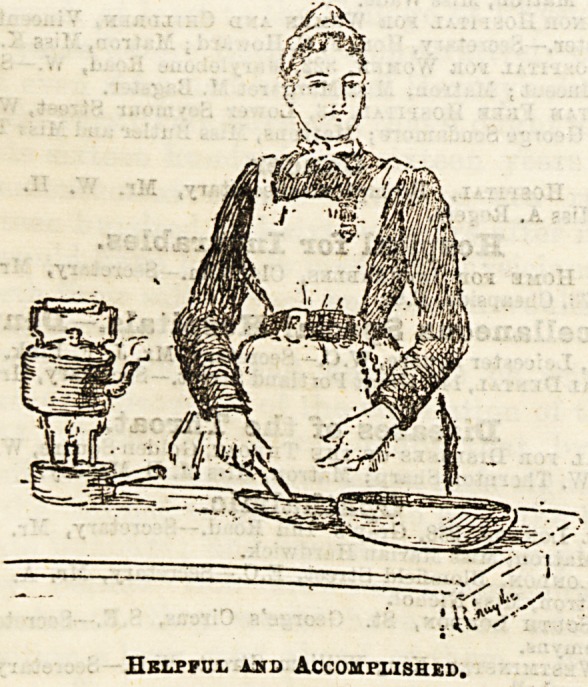


**Figure f5:**
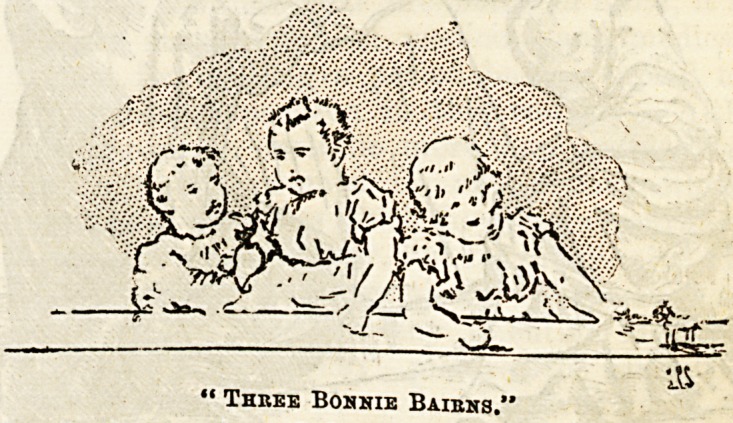


**Figure f6:**
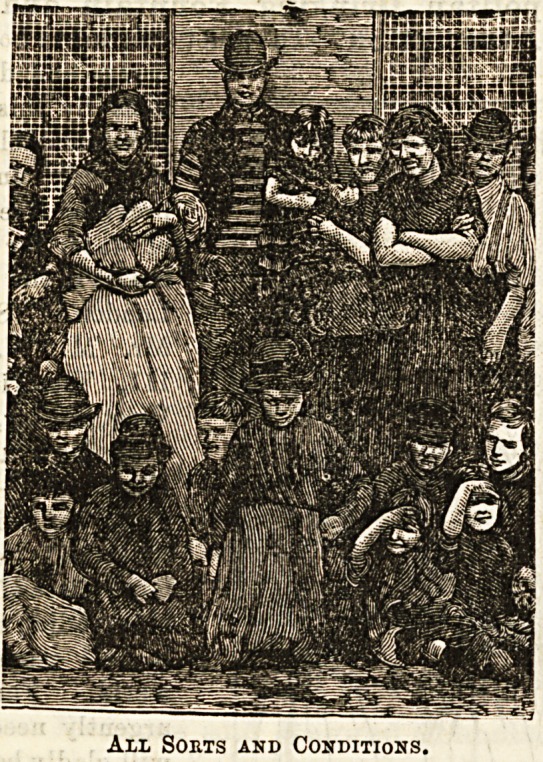


**Figure f7:**